# A single bolus of high dose levothyroxine (L-T_4_) as a test in cases of suspected poor compliance to L-T4 therapy

**DOI:** 10.1186/s13044-015-0028-0

**Published:** 2015-12-01

**Authors:** Krzysztof C. Lewandowski, Katarzyna Dąbrowska, Iwona Komorowska-Dudek, Andrzej Lewiński

**Affiliations:** Department of Endocrinology and Metabolic Diseases, The Medical University of Lodz, Lodz, Poland; Polish Mother’s Memorial Hospital—Research Institute, Lodz, Poland

**Keywords:** Levothyroxine, Adherence, Compliance

## Abstract

**Background:**

Though compliance (or adherence) problems, as well as inappropriate levothyroxine (L-T_4_) intake (e.g. with meal, other drugs or certain foods that can significantly affect absorption) are very common, the issue is often either not mentioned or even frankly denied by patients.

**Case Presentation:**

We describe three cases of patients who presented with high TSH (ranging from about 30 to 200 mIU/l), with concomitantly either high, normal or low free thyroxine (FT_4_), despite treatment with high doses of L-T_4_. The above mentioned problems with adjustment of L-T_4_ dose persisted for several months or even years. Coeliac disease screen was negative in all cases.

In all these patients administration of a single bolus of L-T_4_ (1000 μg) or two doses of 1000 μg of L-T_4_ within 48 h resulted in a quick increase in FT_4_ (thus confirming proper absorption) and in normalization of TSH within a week. No adverse effects of administration of these high doses of L-T_4_ were observed.

**Conclusions:**

Our data support the efficacy, as well as safety of administration of single bolus of high dose L-T_4_ as a test for possible compliance/adherence problems.

## Background

Compliance (also termed adherence) problems, as well as inappropriate intake of levothyroxine (L-T_4_) (e.g. with meal, other drugs or certain foods that affect absorption) are considered to be very common, yet, the issue is typically either not mentioned, or even denied by patients. Precise incidence of compliance problems is not known, but may be quite high. For instance, Morris et al. [1] report that about 40 % of 74 pediatric patients (median age 12.5 years) had at least one episode of non-compliance following thyroidectomy. Here we present three cases, where suspected poor compliance was confirmed by a high dose L-T_4_ test.

### Patient I

A 22-year old male after thyroidectomy (BMI 26.1 kg/m^2^) for poorly controlled Graves’ disease, complicated by recurrent laryngeal nerve palsy and hypocalcaemia (post-surgical hypoparathyroidism) presented with high TSH and high free thyroxine (FT_4_) despite 150 μg of L-T_4_ daily (Table 1). A possibility of poor compliance to medication was raised, particularly in view of episodes of concomitantly high TSH and high FT_4_ (Table 1). He was admitted to hospital, where 1000 μg of L-T_4_ was administered without any side-effects. This was followed by normalization of TSH and FT_4_ (Table 2). Subsequently, he has never turned up for any future follow-up.

**Table 1:** Thyroid function tests of 22-year old male prior to L-T_4_ test

Date	TSH (μIU/ml)	Reference range (μIU/ml)	FT_4_ (ng/dl)	Reference range (ng/dl)	Free triiodothyronine (FT_3_) (pg/ml)	Reference range (pg/ml)
06.01	<0.005^a^	0.27–4.2	1.21^a^	0.93–1.7	7.35	2.6–4.4
18.03	-	0.27–4.2	2.11	0.93–1.7	9.28	2.6–4.4
10.05	-	0.27–4.2	0.583^a^	0.93–1.7	3.98	2.6–4.4
17.06^a^	80.74	0.27–4.2	-	0.93–1.7	-	2.6–4.4
06.09	15.77	0.27–4.2	1.74	0.93–1.7	-	2.6–4.4
23.09	19.22	0.27–4.2	-	0.93–1.7	-	2.6–4.4
03.11	27.84	0.27–4.2	1.42	0.93–1.7	-	2.6–4.4
10.11	14.53	0.27–4.2	1.93	0.93–1.7	3.99	2.6–4.4

^a^results after thyroidectomy in June 2010

**Table 2:** 1000 μg L-T_4_ test in Patient I

Date	TSH (μIU/ml)	Reference range (μIU/ml)	FT_4_ (ng/dl)	Reference range (ng/dl)	FT_3_ (pg/ml)	Reference range (pg/ml)
Day 0^a^	18.92	0.27–4.2	1.54	0.93–1.7	3.06	2.6–4.4
Day 1	8.79	0.27–4.2	4.32	0.93–1.7	4.69	2.6–4.4
Day 2	6.87	0.27–4.2	3.36	0.93–1.7	4.69	2.6–4.4
Day 3	2.34	0.27–4.2	2.58	0.93–1.7	4.26	2.6–4.4
Day 4	1.40	0.27–4.2	2.22	0.93–1.7	3.84	2.6–4.4
Day 5	1.16	0.27–4.2	1.82	0.93–1.7	4.93	2.6–4.4
Day 6	2.76	0.27–4.2	1.65	0.93–1.7	3.45	2.6–4.4

^a^received 1000 μg of L-T_4_ (Euthyrox N®) as a single fasting dose

### Patient II

Twenty-one year old female (weight about 55 kg, BMI 21.2 kg/m^2^) presented with very high TSH (198.76 μIU/ml during hospital admission, previous TSH in the outpatient setting 378 μIU/ml) despite taking 175 μg of L-T_4_. Problems with adequate control dated back about 6 years with massive swings in TSH concentrations (e.g. TSH 92.58 μIU/ml and 23.3 on 200 μg of L-T_4_, and 2 years later TSH 0.062 μIU/ml on 150 μg of L-T_4_). Dilution test (Table 3) showed no evidence of interference, with similar post-dilution recovery concentrations. Levothyroxine (1000 μg) was administered with quick reduction of TSH and evidence of good absorption of L-T_4_ (a rise in FT_4_ from 1.13 ng/dl to 2.72 ng/dl within 10 h) -Table 4. She eventually admitted to missing occasional tablets and more often to taking L-T_4_ with or even after breakfast with morning coffee. About 2 months post discharge she presented with suppressed TSH. The dose of L-T_4_ was gradually decreased to 125 μg/day.

**Table 3:** Serial dilution test of 21 year old female with very high TSH

Date	TSH (μIU/ml)	Dilution 4×	Dilution 8×	Dilution 16×	Dilution 32×
Day 0	>100	198.76 (4 × 48.94)	199.44 (8 × 24.93)	209.8	221.12
			Recovery:	(16 × 13.11)	(32 × 6.91)
			100.3 %	Recovery:	Recovery:
			(199.44 × 100 %/198.76)	105.5 %	111.2 %

**Table 4:** 1000 μg L-T_4_ test in Patient II

Date	TSH (μIU/ml)	Reference range (μIU/ml)	FT_4_ (ng/dl)	Reference range (ng/dl)	FT_3_ (pg/ml)	Reference range (pg/ml)
Day 0	>100	0.27–4.2	1.13	0.93–1.7	2.01	2.6–4.4
Day 0^a^ (6.00 PM)	37.91	0.27–4.2	2.72	0.93–1.7	2.10	2.6–4.4
Day 1	25.54	0.27–4.2	1.99	0.93–1.7	2.96	2.6–4.4
Day 2	10.37	0.27–4.2	1.82	0.93–1.7	3.22	2.6–4.4
Day 3	7.82	0.27–4.2	1.58	0.93–1.7	3.09	2.6–4.4

^a^received 1000 μg of L-T_4_ (Euthyrox N®) as a single fasting dose

### Patient III

Fifty-eight year old obese woman (BMI about 35 kg/m^2^) presented with high TSH and low FT_4_ despite taking high dose of L-T_4_ (300 μg/day). She had a history of thyroidectomy for Graves’s disease about 3 years before, and according to documentation she was rather erratic in taking her methimazole tablets, that was one of the reasons to proceed with thyroid surgery. She also had a history of cholecystectomy for cholelithiasis and was waiting for appointment with gastroenterologists because of abnormal liver function tests (Table 5). She was taking Calcium and vitamin D preparations for osteoporosis prophylaxis. She denied excessive alcohol consumption. Two doses of 1000 μg of L-T_4_ were administered within 48 h without any adverse effects. This was followed by marked improvement of her well-being, as well as her thyroid function tests results (Table 6). She was told that it was essential to take her L-T_4_ tablets at least 60 min before breakfast (that she had not done before). Two months post discharge she had a low-normal FT_4_ [0.97 ng/dl (referenced range 0.93–1.70)] and slightly raised TSH [12.06 mIU/l (reference range 0.27–4.20)]. She did not turn up for her follow-up appointment scheduled 1 week after her blood test.

**Table 5:** Liver function of 58-year old women with high TSH on 300 μg of L-T_4_

Liver function tests	Result (IU/l)	Reference range (IU/l)
ASPAT	182.00	14–36
ALAT	102.00	9–52
GGTP	452.00	12–43
Alkaline phosphatase	87	38–126

**Table 6:** High dose L-T_4_ test in Patient III (1000 μg twice within 48 h)

Date	TSH (μIU/ml)	Reference range (μIU/ml)	FT_4_ (ng/dl)	Reference range (ng/dl)	FT_3_ (pg/ml)	Reference range (pg/ml)
Day 0^a^ (8.00 AM)	18.64	0.27–4.2	0.36	0.93–1.7	0.86	2.6–4.4
Day 0 (6.00 PM)	20.37	0.27–4.2	1.18	0.93–1.7	1.56	2.6–4.4
Day 1	17.43	0.27–4.2	1.08	0.93–1.7	1.72	2.6–4.4
Day 2^a^ (8.00 AM)	15.69	0.27–4.2	2.08	0.93–1.7	1.98	2.6–4.4
Day 3	11.54	0.27–4.2	1.71	0.93–1.7	2.34	2.6–4.4
Day 4	12.60	0.27–4.2	1.58	0.93–1.7	2.31	2.6–4.4
Day 5	8.50	0.27–4.2	1.46	0.93–1.7	2.21	2.6–4.4

^a^ received 1000 μg of L-T_4_ (Euthyrox N®) as a single fasting dose

## Discussion

Our cases demonstrate that administration of a single high dose of L-T_4_ can be useful in cases of suspected poor compliance. Due to a relatively long half-life of L-T_4_ an idea of once-weekly administration regimen had been raised before and tested with a considerable success [2, 3], where single doses of L-T_4_ oscillated around 1000–1500 μg. Though administration of such high dose of L-T_4_ might be thought to be potentially associated with side-effects, such as heart palpitations, angina, etc., the test was reported to be surprisingly well tolerated [4–6]. Furthermore, some patients actually preferred a weekly, rather than a daily L-T_4_ administration [4, 6]. In accordance with these data we did not observe any adverse effects of administration of these high doses of L-T_4_, while some authors (KCL) participated in a study where even a higher dose of L-T_4_ (2000 μg) was administered without any side-effects, while testing for heterophilic antibodies interference [7]. According to the literature data, the upper range of age of patients tested for possible non-adherence with high dose L-T_4_ was as high as 88 years [6]. Also current FDA bioequivalence guidelines require that supra-therapeutic doses of L-T_4_ (around 600 μg) should be administered in studies aiming to assess pharmacokinetics of L-T_4_ preparations [8]. One must be, however, aware that despite an excellent tolerance data, the number of patients participating in weekly L-T_4_ studies was still relatively small (e.g. 12 patients in 3, or 23 patients in 6), so that a caution must be applied in case of patients with a history of active ischaemic heart disease (e.g. confirmed unstable angina, after coronary artery by-pass grafts, etc.).

Another issue pertains to the very definition of non-compliance or non-adherence. In case of L-T_4_, this may involve both missing tablets completely (most likely in Patient I), or taking them tablets inappropriately, e.g. with meal or after meal with concomitant ingestion of coffee, etc. (Patients II and III). For instance, Bach-Huynh et al. [9] demonstrated that even with full compliance, an ingestion of L-T_4_ tablets within 20 min of breakfast results in more than 100 % increase in mean TSH concentrations (1.54 mIU/l *versus* 3.74 mIU/l), as well as a dispersion of measured TSH values up to around 19 mIU/l. Though in a seminal study of Wenzel and Kirschsieper [10] ingestion of L-T_4_ was reduced down to 64 % (for a 100 μg dose), if taken with food, even lower values (down to 40 %) are quoted [8]. Certain food and drinks affect absorption of L-T_4_. In particular this includes dietary fibres (e.g. in muesli, corn-flakes), coffee, grapes, soybeans and papaya fruits [11], of which the first two are commonly consumed with breakfast in Poland. Calcium salts, currently commonly combined with vitamin D, as a part of osteoporosis prophylaxis, and often taken with food during breakfast (applicable in our Patient III), can also significantly impair L-T_4_ absorption [12]. Absorption of L-T_4_ is also decreased in cases of higher gastric pH, such as atrophic gastritis, use of proton pump inhibitors, as well as *Helicobacter pylori* infection [8, 11]. Intestinal, as well as hepatic diseases are associated with worse absorption of L-T_4_. This might be relevant to our Patient III, as she had previous cholecystectomy, abnormal liver function tests and was taking Calcium and vitamin D preparations for osteoporosis prophylaxis. She was referred for gastroenterological consultation with view of performing an endoscopic retrograde cholangiopancreatography (ERCP). There is also a well known phenomenon of enterohepatic circulation of thyroid hormones [13], that was also therapeutically explored for instance for treatment of thyrotoxicosis in humans [14, 15]. In particular, biliary disease might have potentially influenced reabsorption of thyroid hormones, particularly in a hypothyroid state, as there are data [13] that hypothyroid rats had diminished levels of bile acid synthesis and biliary secretion of cholesterol and phospholipid. In such settings, a single dose triiodothyronine (T_3_) injection produced a 13-fold increase in bile cholesterol secretion and a 3-fold increase in phospholipid secretion, both initiated 12 h after T_3_ administration [13]. It should mentioned, however, that though unresolved issue of abnormal liver function might have contributed to a decrease of L-T_4_ absorption in Patient III, it was unlikely to be a sole factor responsible for clinical picture observed in this patient. This is because of a striking increase of FT_4_ concentrations (from 0.36 ng/dl, to 1.18 ng/dl) at about 10 h post ingestion of 1000 μg of L-T_4_ (on an empty stomach with breakfast delayed to 60 min post ingestion). This confirms an adequate absorption of L-T_4_ tablets on condition that they were swallowed in an appropriate fashion. Indeed Walker et al. [6] studied absorption of L-T_4_ (once a week, dose calculated as 1.6 μg/kg per day and given as a single weekend dose) at 60, 120, 180 and 240 min after L-T_4_ bolus, and concluded that in 82 % of cases the maximal rise of FT_4_ occurred as soon as at 120 min. Hence, there is a possibility that in our patient FT_4_ concentrations might be even higher if tested at an earlier timing. It might be also interesting to mention, that Australian authors [16] proposed a variation of L-T_4_ absorption test that involves co-administration of paracetamol tablets for detection of cases of deliberate avoidance of tablet swallowing.

## Conclusions

In summary, our data support the efficacy, as well as safety of administration of single bolus of high dose L-T_4_ in some patients who fail to normalize their thyroid function tests, despite prolonged attempts to adjust their L-T_4_ dose in the outpatient settings. To the best of our knowledge this is the first paper in Polish medical literature describing clinical application of bolus high dose L-T_4_ test for possible non-compliance problems.

### Consent

Written informed consent was obtained from the patients for publication of this case report. A copy of the written consent is available for review by the Editor-in-Chief of this journal.
